# Glyoxal as an alternative fixative for single-cell RNA sequencing

**DOI:** 10.1093/g3journal/jkad160

**Published:** 2023-07-26

**Authors:** Josephine Bageritz, Niklas Krausse, Schayan Yousefian, Svenja Leible, Erica Valentini, Michael Boutros

**Affiliations:** Division Signaling and Functional Genomics, German Cancer Research Center (DKFZ), Im Neuenheimer Feld 580, 69120 Heidelberg, Germany; Division Signaling and Functional Genomics, German Cancer Research Center (DKFZ), Im Neuenheimer Feld 580, 69120 Heidelberg, Germany; Division Signaling and Functional Genomics, German Cancer Research Center (DKFZ), Im Neuenheimer Feld 580, 69120 Heidelberg, Germany; Division Signaling and Functional Genomics, German Cancer Research Center (DKFZ), Im Neuenheimer Feld 580, 69120 Heidelberg, Germany; Division Signaling and Functional Genomics, German Cancer Research Center (DKFZ), Im Neuenheimer Feld 580, 69120 Heidelberg, Germany; Division Signaling and Functional Genomics, BioQuant and Medical Faculty Mannheim, German Cancer Research Center (DKFZ), Heidelberg University, D-69120 Heidelberg, Germany

**Keywords:** single-cell RNA sequencing, transcriptomics, cell fixation, glyoxal

## Abstract

Single-cell RNA sequencing has become an important method to identify cell types, delineate the trajectories of cell differentiation in whole organisms, and understand the heterogeneity in cellular responses. Nevertheless, sample collection and processing remain a severe bottleneck for single-cell RNA sequencing experiments. Cell isolation protocols often lead to significant changes in the transcriptomes of cells, requiring novel methods to preserve cell states. Here, we developed and benchmarked protocols using glyoxal as a fixative for single-cell RNA sequencing applications. Using Drop-seq methodology, we detected a large number of transcripts and genes from glyoxal-fixed *Drosophila* cells after single-cell RNA sequencing. The effective glyoxal fixation of transcriptomes in *Drosophila* and human cells was further supported by a high correlation of gene expression data between glyoxal-fixed and unfixed samples. Accordingly, we also found highly expressed genes overlapping to a large extent between experimental conditions. These results indicated that our fixation protocol did not induce considerable changes in gene expression and conserved the transcriptome for subsequent single-cell isolation procedures. In conclusion, we present glyoxal as a suitable fixative for *Drosophila* cells and potentially cells of other species that allow high-quality single-cell RNA sequencing applications.

## Introduction

The development of single-cell RNA sequencing (scRNA-seq) methods has opened new analytical avenues in the molecular life sciences (Picelli 2017; Aldridge and Teichmann 2020). Genome-wide transcriptomic data are now routinely generated with single-cell resolution on plate- (Picelli *et al.* 2013) or microfluidics-based systems (Klein *et al.* 2015; Macosko *et al.* 2015). Despite a rapidly expanding number of improved technologies, the overall workflow remains similar. Following separation, single cells are lysed in individual reaction chambers. The released mRNA is converted into cDNA libraries, PCR-amplified, and further processed for high-throughput sequencing. Most enhancements of scRNA-seq technologies focus either on different aspects of sequencing library preparation or on downstream analysis tools. However, also single-cell sample preparation is of great importance. Whether working with cell lines or primary tissue, optimizing and tailoring the cell isolation protocol for the specific sample type is important for achieving overall single-cell data quality. The aim of this study is to fully disaggregate the sample into single cells without compromising their viability or integrity, which minimizes the level of free RNA from dead, dying, or damaged cells and thus reduces experimental noise. While scRNA-seq experiments should be carried out without much delay in order to prevent degradation of RNA and thus changes in the transcriptome, in reality, the experimental design often requires flexibility in the sample collection process. Single-nuclei RNA sequencing (nucSeq) from frozen tissue samples offers a suitable way to bypass this constraint. However, the lower transcript counts obtained from nucSeq (Habib *et al.* 2017; Bakken *et al.* 2018) can constitute a drawback of this method, especially when working with model organisms with low RNA content, such as *Drosophila*.

Sample fixation has the potential to preserve the transcriptome and ease the scRNA-seq experiment at the same time. However, despite the highly increasing number of scRNA-seq studies (Svensson *et al.* 2018), protocols with fixed single cells are used only to a limited extent (Thomsen *et al.* 2016; Alles *et al.* 2017; Karaiskos *et al.* 2017; Attar *et al.* 2018; Wohnhaas *et al.* 2019; Denisenko *et al.* 2020; Phan *et al.* 2021; Wang *et al.* 2021). Among the observed disadvantages are a reduced library complexity with a lower number of detected transcripts (Alles *et al.* 2017; Attar *et al.* 2018; Phan *et al.* 2021) and a higher level of ambient RNA (Wohnhaas *et al.* 2019). A comparative analysis of alternative fixation methods, however, has not yet been performed.

Here, we performed glyoxal fixation on *Drosophila* and human cell lines and analyzed their transcriptome using Drop-seq. The gene expression profiles of unfixed and glyoxal-fixed samples showed an overall high similarity for both species. In particular, glyoxal fixation of *Drosophila* cells resulted in high-quality scRNA-seq data with a low fraction of mitochondrial encoded RNA and a large number of detected genes and transcripts. Our glyoxal fixation protocol has the potential to increase both quality and comparability of scRNA-seq data particularly for *Drosophila* samples and other model organisms with low RNA amount.

## Methods

### Cell line fixation for scRNA-seq

Kc167 cells were cultured in Schneider's media supplemented with 10% heat-inactivated fetal calf serum (FCS) at 25°C in T75 cell culture flasks with solid caps. HEK 293T cells were cultured in Dulbecco's Modified Eagle Medium (DMEM) supplemented with 10% FCS and 1% penicillin/streptomycin at 37°C and 5% CO_2_ in T75 cell culture flasks with filter caps. For preparing a single-cell solution, the Kc167 cells were mechanically detached, transferred to a 15 ml tube, and centrifuged at 260 rcf for 5 min at room temperature. The cell pellet was washed once with filtered PBS, resuspended in filtered PBS, and filtered through a 20 µm cell strainer. The HEK 293T cells were washed with filtered PBS (0.22 µm filter) and enzymatically detached with 2 ml 1× TrypLE. The reaction was inactivated with a 10 ml filtered PBS. The detached cells were filtered through a pre-equilibrated 40 µm cell strainer. Cell concentrations were determined with a disposable Neubauer hemocytometer (C-Chip DHC-N01). Unfixed cells were directly processed by scRNA-seq. For glyoxal fixation, 5 × 10^6^ cells per condition and cell line were resuspended in 1 ml 3% glyoxal mix pH 4 (Richter *et al.* 2018) and incubated for 1 h on ice. Next, the fixed cell solution was centrifuged at 260 rcf for 5 min at 4°C, washed twice with filtered PBS, and filtered through a pre-equilibrated 40 µm (HEK 293T) or 20 µm (Kc167) cell strainer. The HEK 293T and Kc167 single cells were equally mixed to a final cell concentration of 100 cells/µl and used for scRNA-seq.

### scRNA-seq by Drop-seq

Single-cell transcriptomic data were generated with Drop-seq following available protocols (Macosko *et al.* 2015; Bageritz, Raddi, *et al.* 2019). In brief, cells and barcoded beads (ChemeGene) were coflowed into a T-junction polydimethylsiloxane microfluidic device (FlowJem) and coencapsulated in 125 μM droplets. High-quality emulsions were broken by perfluorooctanol and reverse transcription of captured polyadenylated mRNA was performed. Subsequently, barcoded beads were incubated with Exonuclease I to remove excess primers, and cDNA was amplified with 14 PCR cycles from 2× 2,000 beads (replicate 1) and 4,000 beads (replicate 2), respectively. A 0.6 ratio of AMPure beads (Agencourt) was used to purify cDNA libraries, which were eluted in 10 μl water. Final libraries were prepared using the Illumina Nextera XT kit. Paired-end sequencing was carried out with the Illumina HiSeq2500 instruments at the DKFZ Genomics and Proteomics Core Facility (Heidelberg, Germany). Experiments were performed in 2 biological replicates. For the first biological replicate, technical replicates were generated during PCR amplification, while the second biological replicate included technical replicates from the unfixed sample from 2 different Drop-seq runs. Individual libraries were prepared for each replicate and sequenced. For downstream analysis, individual technical replicates were merged and analyzed together.

### Processing and quality assessment of scRNA-seq data

Sequencing data were processed as described by Macosko *et al.* (2015). An interface to the used R functions was implemented in our in-house Galaxy server following the default settings described in detail in the Drop-seq computational cookbook v.1.2 accessed at http://mccarrolllab.org/dropseq/ and described in detail (Macosko *et al.* 2015). The reads were aligned to the *Drosophila* reference genome (BDGP6 v.91 (GCA 000001215.4) and GRCh37.87 (hg19)) using STAR v.2.5.2b-0 with the default parameters and showed at least 70% uniquely mapped reads for all samples. The CollectRnaSeqMetrics tool from Picard v2.18.2 (Broad Institute 2018) was used to collect metrics about the alignments. The cell number was estimated by plotting the cumulative fraction of reads per cell against the sorted cell barcodes (decreasing number of reads) and determining the point of inflection. The generated digital gene expression matrices were then further analyzed with R 3.6.3 (using R studio) using the Seurat v3.1.5 package (Satija *et al.* 2015). We kept cells with a minimum of 200 detected genes, analyzed the fraction of mitochondrial encoded RNA, and removed outlier cells from further analysis. The chosen threshold was <5% for the *Drosophila* samples and <10% for the unfixed and <20% for the glyoxal-fixed human sample. The level of the species-mixed cell barcodes was assessed, and cells with <100% purity were assigned as mixed population and discarded from further analysis.

### Data normalization

As samples have been sequenced on 5 flow cell lanes, they showed batch effects mainly originating from differences in sequencing depths. We therefore decided to normalize them with the following steps: First, from each BAM file, we extracted the reads belonging to the real cells as determined from the knee plot and then we randomly sampled a fraction of reads from each extracted BAM file to have the same average number of reads per cell among the different samples. The library with the lowest average number of reads per species was taken as reference. *Drosophila* samples were normalized to 12,697 reads per cell and human samples to 24,791 reads per cell. For both operations, we used pysam, a Python wrapper around the SAMtools package (https://github.com/pysam-developers/pysam, Li *et al.* 2009).

### Evaluation of library complexity

The sequencing saturation level is a measure of the fraction of library complexity that was sequenced in a given experiment and has been calculated for the normalized libraries using the following formula:


Sequencingsaturation=1−(n_deduped_reads/n_reads),

where *n*_deduped_reads is the number of transcripts (UMI), and *n*_reads is the number of reads.

### Sample comparison

To compare the biological replicates and the cells treated with glyoxal and untreated for both human and *Drosophila*, we computed the gene expression correlation of the genes in common. The gene expression was normalized by calculating the average UMI expression for each gene and converting it to average transcript per million (ATPM). We then calculated the Pearson's correlation coefficient (*R*) based on the normalized expression values. Single-cell expression data were aggregated to “pseudobulk” data, and the 100 most expressed genes were compared between unfixed and glyoxal-fixed samples.

## Results

### Effects of glyoxal fixation on scRNA-seq performance

**Fig. 1.**
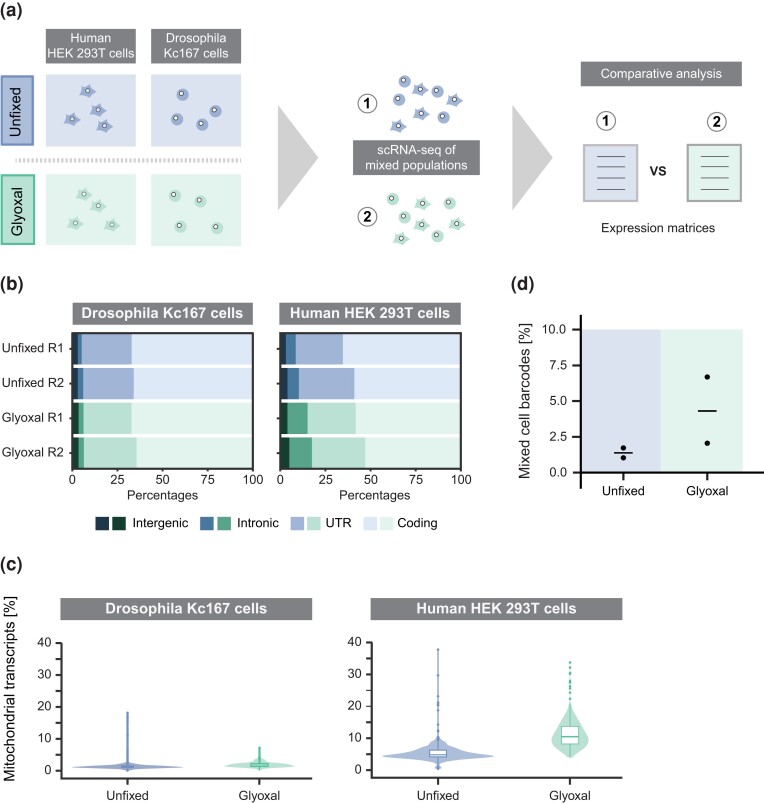
Effect of glyoxal fixation on cell integrity, RNA quality, and purity. a) Workflow to assess the quality of glyoxal-fixed human and *Drosophila* cell lines for single-cell RNA transcriptome studies. Single-cell suspension from different species was mixed equally, subjected to Drop-seq, and their transcriptome compared by downstream analysis. b) Proportion of reads mapped to coding, UTR (untranslated region), intronic, and intergenic regions. Two biological replicates were analyzed per condition for each cell line. c) Fraction of mitochondrial transcripts out of the total mRNA transcripts per condition and cell line. d) Percentage of mixed cell barcodes detected per data set. Horizontal line indicates the mean.

Glyoxal is a dialdehyde used as fixative for immunohistochemistry for many years (Dapson 2007) and was recently shown to present a promising alternative to the commonly used paraformaldehyde (PFA) (Richter *et al.* 2018; Channathodiyil and Houseley 2021). Compared to PFA glyoxal has a lower toxicity, a faster fixation speed and shows little tendency to crosslink under certain pH conditions. Those features also make glyoxal a promising fixative for single-cell transcriptome studies. RNA molecules would be quickly preserved without molecule leakage and impaired purity. It would also allow high amounts of RNA recovery without the need to reverse crosslink the samples. To evaluate glyoxal as fixative for scRNA-seq applications, we performed head-to-head comparison of unfixed (blue) and glyoxal-fixed (green) *Drosophila* (Kc167) and human (HEK 293T) cells. Glyoxal fixation was performed by buffering the solution to pH 4, a condition that inhibits crosslinking (Dapson 2007). As described before (Macosko *et al.* 2015), mixed species populations were subjected to scRNA-seq, and the obtained transcriptomic data were thoroughly compared (Fig. 1a).

In order to preserve the transcriptome of a cell, a fixative has to act fast to prevent loss of cytoplasmic mRNA due to leakage of RNA molecules through pores in the cell membrane. In case of RNA leakage, the relative abundance of intron-containing nascent transcripts of the nuclear compartment would increase due to its more protected location. By analyzing the distribution of the transcript coverage in the *Drosophila* data, we found the average read mapping to distinct transcript regions highly comparable between glyoxal-fixed and unfixed samples (Fig. 1b, left). In contrast, glyoxal fixation of human cells introduced minor changes in the transcript coverage. Here, we found a slight increase in the average read number mapping to intronic regions from 6 to 12%, while the average fraction of mature transcripts (untranslated region, coding) was reduced from 91 to 84% upon glyoxal fixation (Fig. 1b, right). Similarly, determining the fraction of mitochondrial RNA within each cell can be used as an indicator for the loss of cytoplasmic RNA in cells (Ilicic *et al.* 2016). In *Drosophila* cells, the proportion of mitochondrial RNA detected in unfixed and glyoxal-fixed samples was again comparable (Fig. 1c, left). A median of <2% mapped mitochondrial transcripts has been found in both, unfixed and glyoxal-fixed cells. In contrast, glyoxal fixation of human cells led to an increase in mapped mitochondrial transcripts with a median fraction of 4.8% in unfixed and 10.5% in glyoxal-fixed cells (Fig. 1c, right). Next, we assessed the single-cell purity of the samples and obtained a similar ratio of mixed transcriptomes between unfixed and glyoxal-fixed cells (Fig. 1d). Low level of ambient RNA is apparent in the overlaid kneeplots (Supplementary Fig. 1a), showing a comparable fraction of reads in unfixed and glyoxal-fixed cells, while empty droplets only captured little amount of ambient RNA.

Taken together, we show that glyoxal fixation is compatible with downstream scRNA-seq methodology and that RNA molecules seem to be better fixed by glyoxal in *Drosophila* cells than they are in human cells.

### Preservation of cytoplasmic RNA molecules upon glyoxal fixation

Next, we examined the impact of glyoxal fixation on cytoplasmic mRNA transcripts. We observed a shorter average cDNA size for the species-mixed glyoxal libraries compared to the unfixed samples (Supplementary Fig. 1b), which indicates RNA degradation and/or fragmentation in *Drosophila* and/or human cells. To further assess the effect of RNA leakage and degradation/fragmentation, we analyzed the level of detected genes and transcripts. For this purpose, we normalized the data for differences in sequencing depth (Supplementary Fig. 2a) and found the samples to have a mean library saturation of about 50–70% (Supplementary Fig. 2b). Notably, the unfixed and glyoxal-fixed *Drosophila* samples showed a similar mean saturation level, while the human glyoxal sample showed an almost 20% higher mean saturation than the unfixed human samples indicating lower library complexity. Regardless of the species or experimental condition, the biological replicates correlate well (*R* ≥ 0.88 for *Drosophila* and human data, Supplementary Fig. 2c) allowing us to thoroughly compare the experimental conditions.

**Fig. 2.**
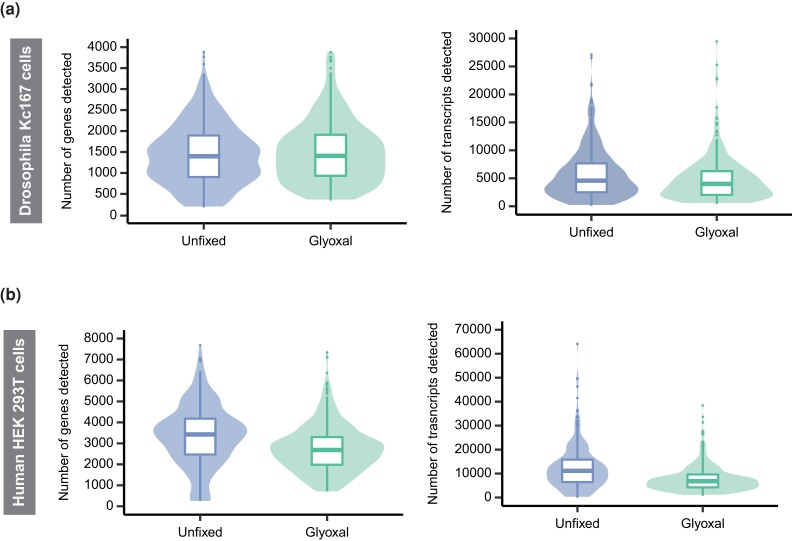
Library complexity in unfixed and glyoxal-fixed samples. a and b) Number of detected genes (left) and transcripts (right) for unfixed and glyoxal-fixed single-cell solutions obtained from *Drosophila* (a) or human (b) cell lines. Two biological replicates were analyzed per condition and cell line. Box plots show median and 25th and 75th percentiles.

In *Drosophila*, the detected gene and transcript counts show a similar distribution in fixed and unfixed cells (Fig. 2a). The median recovery of genes and transcripts for unfixed (1,398 genes/cell and 4,577 transcripts/cell) and glyoxal-fixed (1,408 genes/cell and 4,000 transcripts/cell) are highly comparable. In contrast, the human sample showed a lower variation in detected gene and transcript expression and a reduction in the median number of genes and transcripts upon glyoxal fixation (Fig. 2b). In unfixed human cells, we detected a median of 3,426 genes/cell and 11,142 transcripts/cell, whereas in glyoxal-fixed cells that number was reduced to 2,684 genes/cell and 6,812 transcripts/cell, respectively.

In summary, our data suggest that glyoxal effectively preserves RNA molecules of *Drosophila* cells, while human cells seem to be more prone to cytoplasmic RNA leakage and RNA degradation and/or fragmentation resulting in lower number of detected genes and transcripts.

### Glyoxal fixation in *Drosophila* and human cells preserves the mRNA

**Fig. 3.**
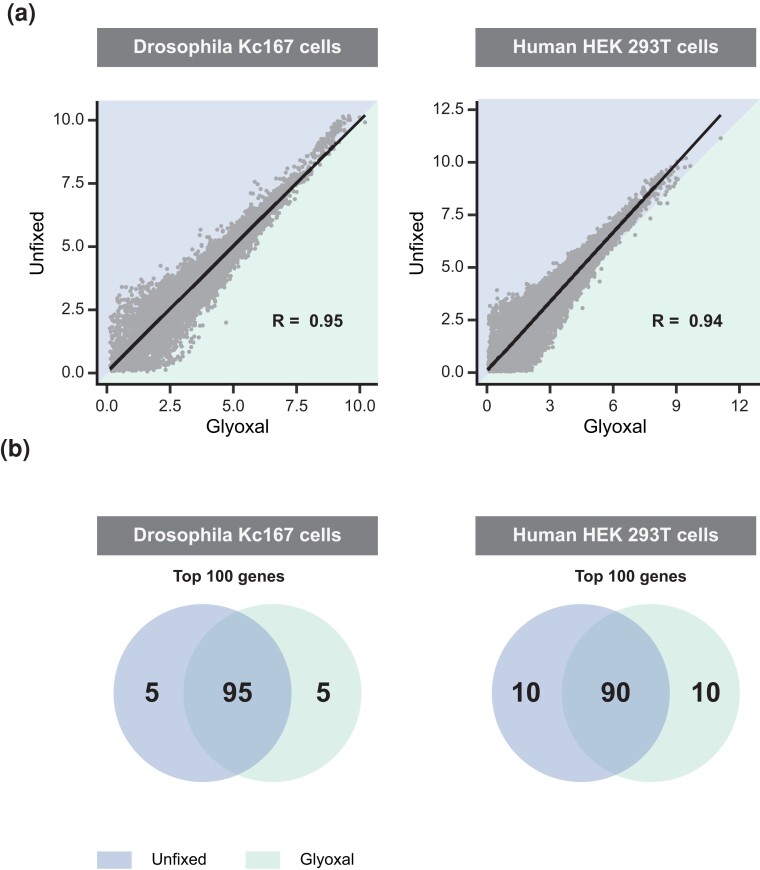
Transcriptome similarities of unfixed and glyoxal-fixed *Drosophila* and human cells. a) High average gene expression correlation between unfixed and glyoxal-fixed libraries. Average gene expression correlation of shared genes between pooled unfixed and glyoxal-fixed *Drosophila* and human cell lines. Data are shown as normalized ATPM. R indicates the Pearson correlation coefficient. b) Overlap between top 100 highly expressed genes for unfixed and glyoxal-fixed *Drosophila* (left) and human (right) cell lines depicted in Venn diagrams.

In order to examine the effect of glyoxal fixation on the entire transcriptome, we aggregated single cells to “pseudobulk” data and compared their gene expression profiles (Fig. 3a). By analyzing the set of commonly expressed genes (8,040 genes in *Drosophila* cells, 17,092 genes in human cells), we showed a high correlation between unfixed and glyoxal-fixed samples (*R* = 0.95 for the *Drosophila* data, *R* = 0.94 for the human data). This allows the conclusion that the transcriptomes of unfixed and glyoxal-fixed *Drosophila* and human cells are highly similar when analyzed as “pseudobulk” data sets. We then extracted the top 100 highly expressed genes from both conditions and determined their overlap (Fig. 3b, Supplementary Table 1). Comparing the unfixed and glyoxal-fixed samples showed with 95 genes in *Drosophila* and 90 genes in human a high overlap, which is similarly seen by comparing the respective technical replicates (Supplementary Fig. 3, Supplementary Table 2) indicating that glyoxal fixation does not change gene expression profiles substantially.

Taken together, in this report, we have identified glyoxal as a suitable fixative for scRNA-seq applications. We found glyoxal fixation to be compatible with high-throughput single-cell sequencing with fixed samples maintaining a preserved transcriptome of high purity, and library complexity.

## Discussion

Glyoxal has successfully been used as fixative for detecting single mRNA molecules by in situ stainings and also the applicability to perform bulk RNA sequencing has been shown (Richter *et al.* 2018; Channathodiyil and Houseley 2021). Here, we report the additional use of glyoxal as fixative for scRNA-seq. Although, previous studies have presented different fixatives for scRNA-seq applications (Thomsen *et al.* 2016; Alles *et al.* 2017; Karaiskos *et al.* 2017; Attar *et al.* 2018; Phan *et al.* 2021), we have found glyoxal fixation to be highly advantageous in many key aspects when it comes to the preservation of transcriptomic data.

Generally, in scRNA-seq experiments, only a small fraction of the transcriptome is captured and so downstream analysis can be quite challenging. High number of detected genes and transcripts is hence aimed for, especially in tissues or model organism with lower number of detected mRNA transcripts in scRNA-seq experiments such as *Drosophila* (Karaiskos *et al.* 2017; Bageritz, Willnow, *et al.* 2019). In contrast to other fixatives, such as methanol, glyoxal fixation did not compromise the level of detected transcripts and genes in *Drosophila* Kc167 cells. Library complexity in human HEK 293T cells was reduced upon glyoxal fixation but not to an extent seen for nucSeq experiments (Habib *et al.* 2017) and to a similar level than methanol fixation (Alles *et al.* 2017; Chen *et al.* 2018). Differences in fixation speed impacting RNA leakage from the cytoplasm and RNA degradation most likely explain the observed quantity loss in human cells. Titrating the pH of the glyoxal mix as previously done for optimizing the immunostaining protocols (Richter *et al.* 2018), and experimentally testing for the optimal fixation time might overcome this limitation for scRNA-seq experiments. Importantly, glyoxal fixation in both human and *Drosophila* cells yields transcriptome data similar to that from unfixed cells with technical noise greater than any effect of glyoxal. This also indicates that glyoxal fixation did not compromise transcript accessibility for library preparation and sequencing.

Isolating single cells from solid tissue can be challenging as dissociation protocols need to be optimized to avoid cell damage and the resulting increased levels of free RNA while at the same time facilitating unbiased recovery of single cells in sufficient quantity. Fixation of the tissue prior to single-cell dissociation could solve this problem. However, there is currently no fixative available that can be efficiently used for tissue fixation prior to the application of any lengthy dissociating protocols. In this regard, it will be interesting to analyze the single-cell recovery of glyoxal-fixed primary tissue. Glyoxal might additionally be useful to uncouple sample collection from further processing by allowing sample storage. This can be useful for clinical samples or in experiments that require a large amount of starting material. Another beneficial aspect would be the utilization of GFP expression for FACS sorting prior to scRNA-seq to enrich certain cell populations. However, these aspects require further experiments and comprehensive testing. Overall, the data presented here highlight glyoxal as a suitable fixative for scRNA-seq experiments. Improved sample preservation will aid in the generation of high-quality data sets for scRNA-seq. However, considering the widespread use of methanol as a fixative for scRNA-seq experiments, conducting benchmarking experiments within one experimental set-up to compare the effectiveness of both methanol and glyoxal as fixatives would be valuable for future studies.

By comparing our results with available published scRNA-seq data of fixed samples, we present with glyoxal a comparably cheap, easy-to-handle, and most of all, effective fixative, which can be easily implemented in existing experimental work flows.

## Supplementary Material

jkad160_Supplementary_Data

## Data Availability

The generated scRNA-seq count matrices are deposited in the Gene Expression Omnibus database and are accessible through accession number GSE163736. In our GitHub repository (https://github.com/boutroslab/Supp_Bageritz_2023), you will find both the code and a description of the required input files to normalize the scRNA-seq data. The top 100 highly expressed genes for unfixed and glyoxal-fixed *Drosophila* and human cell lines are provided in Supplementary Tables 1 and 2. Supplemental material available at G3 online.

## References

[jkad160-B1] Aldridge S , Teichmann SA. Single cell transcriptomics comes of age. Nat Commun. 2020;11(1):4307. doi:10.1038/s41467-020-18158-5. 32855414 PMC7453005

[jkad160-B2] Alles J , Karaiskos N, Praktiknjo SD, Grosswendt S, Wahle P, Ruffault P-L, Ayoub S, Schreyer L, Boltengagen A, Birchmeier C, et al Cell fixation and preservation for droplet-based single-cell transcriptomics. BMC Biol. 2017;15(1):44. doi:10.1186/s12915-017-0383-5. 28526029 PMC5438562

[jkad160-B3] Attar M , Sharma E, Li S, Bryer C, Cubitt L, Broxholme J, Lockstone H, Kinchen J, Simmons A, Piazza P, et al A practical solution for preserving single cells for RNA sequencing. Sci Rep. 2018;8(1):2151. doi:10.1038/s41598-018-20372-7. 29391536 PMC5794922

[jkad160-B4] Bageritz J , Raddi G, Proserpio V. Single-cell RNA sequencing with Drop-seq. In: Proserpio V, editor. Single Cell Methods: Sequencing and Proteomics. New York (NY): Springer; 2019. p. 73–85. 10.1007/978-1-4939-9240-9_6 31028633

[jkad160-B5] Bageritz J , Willnow P, Valentini E, Leible S, Boutros M, Teleman AA. Gene expression atlas of a developing tissue by single cell expression correlation analysis. Nat Methods. 2019;16(8):750–756. doi:10.1038/s41592-019-0492-x. 31363221 PMC6675608

[jkad160-B6] Bakken TE , Hodge RD, Miller JA, Yao Z, Nguyen TN, Aevermann B, Barkan E, Bertagnolli D, Casper T, Dee N, et al Single-nucleus and single-cell transcriptomes compared in matched cortical cell types. PLoS One. 2018;13(12):e0209648. doi:10.1371/journal.pone.0209648. 30586455 PMC6306246

[jkad160-B7] Broad Institute . Picard tools. 2018. http://broadinstitute.github.io/picard/.

[jkad160-B8] Channathodiyil P , Houseley J. Glyoxal fixation facilitates transcriptome analysis after antigen staining and cell sorting by flow cytometry. PLoS One. 2021;16(1):e0240769. doi:10.1371/journal.pone.0240769. 33481798 PMC7822327

[jkad160-B9] Chen J , Cheung F, Shi R, Zhou H, Lu W; CHI Consortium. PBMC fixation and processing for Chromium single-cell RNA sequencing. J Transl Med. 2018;16(1):198. doi:10.1186/s12967-018-1578-4. 30016977 PMC6050658

[jkad160-B10] Dapson RW . Glyoxal fixation: how it works and why it only occasionally needs antigen retrieval. Biotech Histochem. 2007;82(3):161–166. doi:10.1080/10520290701488113. 17987441

[jkad160-B11] Denisenko E , Guo BB, Jones M, Hou R, de Kock L, Lassmann T, Poppe D, Clément O, Simmons RK, Lister R, et al Systematic assessment of tissue dissociation and storage biases in single-cell and single-nucleus RNA-seq workflows. Genome Biol. 2020;21(1):130. doi:10.1186/s13059-020-02048-6. 32487174 PMC7265231

[jkad160-B12] Habib N , Avraham-Davidi I, Basu A, Burks T, Shekhar K, Hofree M, Choudhury SR, Aguet F, Gelfand E, Ardlie K, et al Massively parallel single-nucleus RNA-seq with DroNc-seq. Nat Methods. 2017;14(10):955–958. doi:10.1038/nmeth.4407. 28846088 PMC5623139

[jkad160-B13] Ilicic T , Kim JK, Kolodziejczyk AA, Bagger FO, McCarthy DJ, Marioni JC, Teichmann SA. Classification of low quality cells from single-cell RNA-seq data. Genome Biol. 2016;17(1):29. doi:10.1186/s13059-016-0888-1. 26887813 PMC4758103

[jkad160-B14] Karaiskos N , Wahle P, Alles J, Boltengagen A, Ayoub S, Kipar C, Kocks C, Rajewsky N, Zinzen RP. The *Drosophila* embryo at single-cell transcriptome resolution. Science. 2017;358(6360):194. doi:10.1126/science.aan3235. 28860209

[jkad160-B15] Klein AM , Mazutis L, Akartuna I, Tallapragada N, Veres A, Li V, Peshkin L, Weitz DA, Kirschner MW. Droplet barcoding for single-cell transcriptomics applied to embryonic stem cells. Cell. 2015;161(5):1187–1201. doi:10.1016/j.cell.2015.04.044. 26000487 PMC4441768

[jkad160-B16] Li H , Handsaker B, Wysoker A, Fennell T, Ruan J, Homer N, Marth G, Abecasis G, Durbin R; 1000 Genome Project Data Processing Subgroup. The Sequence Alignment/Map format and SAMtools. Bioinformatics. 2009;25(16):2078–2079. doi:10.1093/bioinformatics/btp352. 19505943 PMC2723002

[jkad160-B17] Macosko EZ , Basu A, Satija R, Nemesh J, Shekhar K, Goldman M, Tirosh I, Bialas AR, Kamitaki N, Martersteck EM, et al Highly parallel genome-wide expression profiling of individual cells using nanoliter droplets. Cell. 2015;161(5):1202–1214. doi:10.1016/j.cell.2015.05.002. 26000488 PMC4481139

[jkad160-B18] Phan HV , van Gent M, Drayman N, Basu A, Gack MU, Tay S. High-throughput RNA sequencing of paraformaldehyde-fixed single cells. Nat Commun. 2021;12(1):5636. doi:10.1038/s41467-021-25871-2. 34561439 PMC8463713

[jkad160-B19] Picelli S . Single-cell RNA-sequencing: the future of genome biology is now. RNA Biol. 2017;14(5):637–650. doi:10.1080/15476286.2016.1201618. 27442339 PMC5449089

[jkad160-B20] Picelli S , Björklund ÅK, Faridani OR, Sagasser S, Winberg G, Sandberg R. Smart-seq2 for sensitive full-length transcriptome profiling in single cells. Nat Methods. 2013;10(11):1096–1098. doi:10.1038/nmeth.2639. 24056875

[jkad160-B21] Richter KN , Revelo NH, Seitz KJ, Helm MS, Sarkar D, Saleeb RS, D’Este E, Eberle J, Wagner E, Vogl C, et al Glyoxal as an alternative fixative to formaldehyde in immunostaining and super-resolution microscopy. EMBO J. 2018;37(1):139–159. doi:10.15252/embj.201695709. 29146773 PMC5753035

[jkad160-B22] Satija R , Farrell JA, Gennert D, Schier AF, Regev A. Spatial reconstruction of single-cell gene expression data. Nat Biotechnol. 2015;33(5):495–502. doi:10.1038/nbt.3192. 25867923 PMC4430369

[jkad160-B23] Svensson V , Vento-Tormo R, Teichmann SA. Exponential scaling of single-cell RNA-seq in the past decade. Nat Protoc. 2018;13(4):599–604. doi:10.1038/nprot.2017.149. 29494575

[jkad160-B24] Thomsen ER , Mich JK, Yao Z, Hodge RD, Doyle AM, Jang S, Shehata SI, Nelson AM, Shapovalova NV, Levi BP, et al Fixed single-cell transcriptomic characterization of human radial glial diversity. Nat Methods. 2016;13(1):87–93. doi:10.1038/nmeth.3629. 26524239 PMC4869711

[jkad160-B25] Wang X , Yu L, Wu AR. The effect of methanol fixation on single-cell RNA sequencing data. BMC Genomics. 2021;22(1):420. doi:10.1186/s12864-021-07744-6. 34090348 PMC8180132

[jkad160-B26] Wohnhaas CT , Leparc GG, Fernandez-Albert F, Kind D, Gantner F, Viollet C, Hildebrandt T, Baum P. DMSO cryopreservation is the method of choice to preserve cells for droplet-based single-cell RNA sequencing. Sci Rep. 2019;9(1):10699. doi:10.1038/s41598-019-46932-z. 31337793 PMC6650608

