# Supplementary material for: Glyoxal as an alternative fixative for single-cell RNA sequencing
Source: G3 (Bethesda). 2023 Jul 26;13(10):jkad160. doi: 10.1093/g3journal/jkad160 (PMC10542564; doi:10.1093/g3journal/jkad160)
Supplement: jkad160_Supplementary_Data [file jkad160_supplementary_data.zip › Supplementary_table_caption_G3-2023-404239.pdf]

Supplementary table caption

Supplementary table 1: Lists of the top 100 highly expressed genes in the ‘pseudobulk’ data sets of unfixed and glyoxal fixed Drosophila Kc167 cells and human HEK 293T cells. The first tab, labeled "Top100 genes," provides the merged replicate samples' lists, while the second tab, labeled "Top100 genes replicates," lists the genes of the individual technical replicate samples.
